# Female Resistance to Invading Males Increases Infanticide in Langurs

**DOI:** 10.1371/journal.pone.0018971

**Published:** 2011-04-22

**Authors:** Baoping Ren, Dayong Li, Xinmin He, Junhua Qiu, Ming Li

**Affiliations:** 1 Key Laboratory of Animal Ecology and Conservation Biology, Institute of Zoology, Chinese Academy of Sciences, Beijing, China; 2 College of Life Sciences, China West Normal University, Nanchong, Sichuan, China; 3 The Baimaxueshan National Nature Reserve, Diqing Prefecture, Yunnan, China; 4 Shanghai Wild Animal Park, Nanhui District, Shanghai, China; University of Zürich, Switzerland

## Abstract

**Background:**

Infanticide by adult male occurs in many mammalian species under natural conditions, and it is often assumed to be a goal-directed action and explained predominately by sexual selection. Motivation of this behavior in mammals is limitedly involved.

**Methodology and Principal Findings:**

We used long-term reproductive records and direct observation in captivity and in the field of two snub-nosed langur species on the basis of individual identification to investigate how infanticide happened and to be avoided in nonhuman primates. Our observations suggested that infanticide by invading males might be more accidental than goal-directed. The invading male seemed to monopolize all the females including lactating mothers during takeovers. Multiparous mothers who accepted the invading male shortly after takeovers avoided infanticide in most cases. Our results conjectured primiparous mothers would decrease infanticidal possibility if they sexually accepted the invading male during or immediately after takeovers. In the studied langur species, voluntary abortion or mating with the invading male was evidently adopted by females to limit or avoid infanticide by takeover males.

**Conclusions and Significance:**

The objective of the invading male was to monopolize all adult females after his takeover. It appeared that the mother's resistance to accepting the new male as a mating partner was the primary incentive for infanticide. Motivation analysis might be helpful to further understand why infanticide occurs in primate species.

## Introduction

Infanticide occurs in many mammalian species, including rodents, carnivores, pinnipeds and primates, under natural conditions [1]. In primates, infanticide is often associated with male takeovers in one male-multifemale unit [1]–[2]. Current hypotheses predict that infanticide occurs before and during takeovers as a goal-directed action [2]–[3], with sexual selection and social pathology most often cited to explain this behavior. Sexual selection hypothesis does not, however, explain why infanticide occurs rarely and why many infants survive takeovers. The role of infanticide in mammalian social systems is controversial due to our poor understanding of infanticide frequency and motivation, and the evolutionary consequences for the victims and perpetrators [4].

Primate infanticide was first observed in the hanuman langur (*Semnopithecus entellus*) and has been explained by five different hypotheses [3]. Colobine snub-nosed langurs (*Rhinopithecus* spp.) are closely related to hanuman langurs. The females breed seasonally [5]–[6] but remain sexually receptive throughout the year except during the peripartum period [5]. *Rhinopithecus* species tend to form large groups consisting of several one-male units (OMU) and a male can only mate within his OMU [7]–[8]. When a male takeover occurs, most females stay within their OMU [7], [9], although a few may transfer into another [9]. Male juveniles are ousted from the OMU into the group's all-male unit. Establishing an OMU to access fertile females is the ultimate goal for a bachelor. The OMU grouping pattern facilitates infanticide during a male takeover [2]. Consequently, it would be expected that infanticide is relatively common in snub-nosed langurs. To date, however, only a few infanticidal cases have been observed in *R. roxellana* [10] and *R. bieti* [11] species. In addition, no research has yet interpreted infanticidal behavior in any *Rhinopithecus* species. Here we provide our observations on two langur species (*Rhinopithecus* spp.) to try to understand nonhuman primate infanticide by motivation analysis on the basis of individual recognition.

## Methods

### Study animals

Snub-nosed langurs were selected for study as 1) they are colobines and might risk high infanticide by males [3]; 2) they form large cohesive social groups with many OMUs and one AMU; 3) OMU takeovers occur frequently by AMU males [8]; 4) the OMU is unstable during the first few days before the new male monopolizes the remaining females [7]; and 5) sexual interactions between the invading male and resident females are crucial for OMU stabilization [7]–[8]. Here monopolization of females means 1) residential female sexually solicited the invading male voluntarily and 2) the invading male sexually mounted the adult females successfully. When all adult females in the invading OMU sexually accepted the takeover male, the OMU was regarded to be established.

### Study sites and methods

Behavioral observations of *R. roxellana* were conducted from 7:30 to 16:30 at the Shanghai Wild Animal Park, China, during 1995–2001. *Ad libitum* and focal animal sampling were used to record behavioral data. Each monkey in this captive group (also called the exhibition band) of seventeen individuals was individually identified [5], [7]. Reproductive records of a small captive all-female band were summarized to analyze effects of breeding male replacement (Table S1). Date and time that females' sexually accepted the new males were recorded when takeover or breeding male introduction occurred. Voluntary abortion was found in captive *R. roxellana* and defined as aborted fetuses were found and the aborted females did not show apparent pathological change compared with females reproduced at the same year (Veterinarians' diagnosis of the Park).

Behavioral observations of *R. bieti* were conducted at Xiangguqing (99°18′E, 27°36′N) in Baimaxueshan Nature Reserve in Yunnan, China. From May 2008, the study group consisted of 89 individuals belonging to eight OMUs and one AMU. Monkeys were followed from 7:00 to 17:00 almost daily due to inclement weather and inaccessible terrain from May 2008 to February 2010. Some members were individually identified. When a male takeover occurred, the OMU was observed until all adult females sexually accepted the invading male [5], [7].

All statistic tests were performed in SPSS 15.0 and significant level was set at 0.05. Chi-squared test was used to detect differences among frequency data and t-test was used to detect among time data. Average data were presented as mean ±S.D(Standard deviation ).

## Results

### Artificial takeovers and their influence on females' reproduction in captive *R. roxellana*

We collected all reproductive records of the captive *R. roxellana* group from 1995–2001 at Shanghai Wild Animal Park (Table S1). Voluntary abortion occurred more frequently in the all-female band (eight times by all the three females, Suppl. data) than the exhibition band (never observed) (Chi-squared test: χ^2^ = 8, df = 1, P<0.01). Each artificial introduction of a breeding male was designated as a takeover, with both bands experiencing four takeovers.

Throughout the study, invading males approached the target females and passively accepted their sexual solicitation. No attack (forcible or sexual) on adult females was recorded. Newly invading males required a longer period of time (8±3 days, N = 9) than ex-males (4±2days, N = 21) before gaining sexual acceptance from the resident females (t-test t = 9.704, df = 29, *P*<0.01) during OMU stabilization. It took about six days (6±3days, n = 30) for an invading male to monopolize three females.

### Temporary removal of a captive *R. roxellana* infant after breeding male introduction

In 1995, an all-female band (AFB) was formed at Shanghai Wild Animal Park due to space limitation. By 1999, the band included three adult females and two female juveniles. A breeding male was introduced in July 2000 and an OMU was formed thereafter. The zookeeper introduced a breeding male into the AFB for reproductive purpose in July of each year and then removed him after November when the mating season was over (Table S1). The female (Xx) conceived and aborted five times (Four aborted embryos were collected) from 1997–2000 and produced a healthy baby in 2001.

In July 1997, a father was re-introduced to the group when his daughter (named 97-2#) was 42 days old. The mother avoided him for the first three days after he was reintroduced, while both other females copulated with the male during the first two days. After several days, 97-2# developed a sudden acute cough in the morning and was isolated for a medical check. Only 33 minutes after the removal of 97-2#, the mother sexually solicited the father. When the infant was returned two hours later, the mother received her immediately. Observations of the group showed that following the temporary infant-mother separation, the mother no longer avoided the new adult male. She continued lactating until August 1998 and was pregnant again the following mating season (September–November of 1998).

### Takeover and its implication on infanticide in a wild population of *R. bieti*

**Figure 1:**
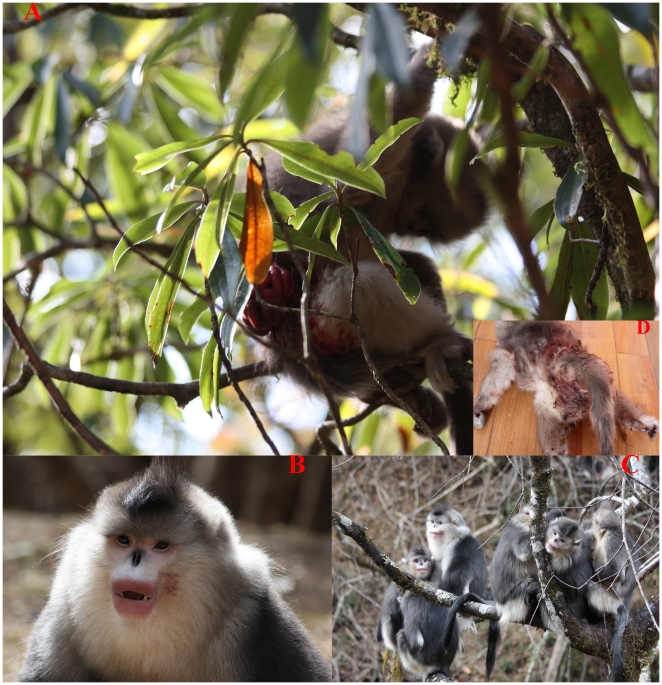
An OMU takeover of *Rhinopithecus bieti* occurred on Dec. 4, 2009. The invading male (B: dry blood on his left cheek) killed the eight-month-old infant (A) at 13:00 on Dec. 31, and the mother sexually solicited the invading male at 14:30. All members of the OMU flocked together (C) in the same tree thereafter. (D) Corpse of the killed infant.

An adult male (XSH) invaded the observed OMU on December 4, 2009 and ousted the resident male into the AMU. The overthrown male died several days later from severe injuries. One eight-month-old male infant and two female juveniles with their mothers stayed in the OMU, while a sub-adult female transferred to another OMU (XM) shortly after the takeover (Table S2). XSH ran to the lactating mother suddenly and the female fled herself without taking away the baby at 7:08 on December 31, 2009. After XSH held the single infant in one hand, the mother and other females jumped on XSH in union abruptly. The male fled with the infant dropping down from his hand, and XSH tried to grasp it but wounded the baby with his reaching hand in the abdomen. The mother picked up the baby from a branch and sat with others of the OMU. XSH sat alone around the victim OMU. The infant was abandoned at 9:30 by his mother in a *Rhododendron* tree (Fig. 1) and he died at 13:00 that same day. The infant-deprived mother was observed voluntarily soliciting the male at 14:43. After mating, all females and juveniles sat together with the male of the OMU. The OMU was established and the male spent 28 days monopolizing all remaining females in the OMU.

Three females transferred from XM OMU to form a new OMU (JJ) on January 18, 2010.

## Discussion

Similar to other nonhuman primates [2], infanticide rarely occurred in snub-nosed langurs (*Rhinopithecus* spp.). Only five infanticide cases have been recorded in wild *R. bieti* (twice [11], and once in this study) and captive *R. roxellana* (twice) [10]. Our findings showed that invading males did not immediately kill suckling infants after takeovers or introduction but attempt to monopolize the mother first. Evidently, infanticide in the two snub-nosed langurs was not goal-directed but seemingly dispensable. Similar to other langurs [12], female snub-nosed langurs are reluctant to sexually accept a new male [5] in the presence of suckling infants.

Theoretically, takeover males should monopolize females in the targeted OMU as much as possible to discourage departure and establish his OMU quickly. From our observations, the monopolizing period was seemingly less than 20days (infanticide occurred after 25 days in *R. roxellana* [10] and 28 days in *R. bieti* [this study]). Primiparous mothers appeared to more forcefully resist sexual acceptance of invading males than multiparous females, which was likely due to infant protection. Killing or temporally removing the suckling infant quickly eliminated the mother's sexual resistance. We assumed that the mothers of infanticide victims may be primiparous females who were unable to correctly deal with takeover males.

Many mammalian females have been found to use aggression, coalition with other males, promiscuity, and reproductive synchrony to confuse paternity as counter-strategies to infanticide [4]. Female snub-nosed langurs appeared to use mating with the invading male in this way. Residential females sexually accepted males they had previously mated with more rapidly than newly invading males [5]. We confirmed that nursing females refused to sexually accept invading males as a protective measure against unfamiliar males rather than due to lactational amenorrhea. Female snub-nosed langurs have, indeed, been known to perform sexual behavior while lactating [5], [8]. In addition, from our study the temporary removal and return of an infant revealed that the mother initially rejected the new male to protect her offspring and did not lack sexual receptivity. The male never attacked the infant following its return. Since consorting females were not attacked, attacks on mother-infant dyads [10] are likely a monopolizing process. In the wild group of *R. bieti*, the mother sexually solicited the new male five hours after the infant was abandoned. The mother then remained close to other OMU members, and the invading male no longer displayed aggressive herding behavior towards her.

We believed it likely that abortion in captive *R. roxellana* females was adopted due to changing breeding males. This indicates that 1) Xx did not suffer habitual abortion and seemingly aborted voluntarily to deal with the unstable OMU consorting male; and 2) *R. roxellana* females can conceive each year and shorten interbirth intervals. Female snub-nosed langurs might, therefore, play a role in infanticidal cases, too.

Only limited data was obtained due to the rarity of snub-nosed langur infanticide. Consequently, it was difficult to determine the evolutionary basis for this behavior. If infanticide was the sole objective, then the sexual selection hypothesis is supported [3]. If infanticide is goal-directed [2], infanticide is then probably a by-product of the sexual selection hypothesis. But in our study, infanticide in snub-nosed langurs occurred incidentally. Additionally, abortion was more frequent than infanticide by invading males in *R. roxellana*; most infants survived under OMU takeovers. The social pathology hypothesis is, therefore, supported infanticide as social pathological behavior is infrequent in animal species due to natural selection [2].

## Supporting Information

Table S1 (DOC)

Table S2 (DOC)
