# Supplementary material for: Female Resistance to Invading Males Increases Infanticide in Langurs
Source: PLoS One. 2011 Apr 22;6(4):e18971. doi: 10.1371/journal.pone.0018971 (PMC3081324; doi:10.1371/journal.pone.0018971)
Supplement: Table S1 — (DOC) [file pone.0018971.s001.doc]

**Table 1. Overview of breeding male introduction and reproductive results of caged Sichuan snub-nosed monkeys at Shanghai Wild Animal Park in** 1996-2001.

| Introduced date | Breeding male | Female reproduction | Notes |
| --- | --- | --- | --- |
| 1996.7.10 | Xs | Xs was accepted by the three females (Tt, Qq, and Xx) after 8 days by active sexual solicitation. | Xs was removed in late November, 1996 and left the all-female band alone. |
| 1997.7.11 | Xs | Xx produced a female 97-2# on May 31 and was nursing the baby. She did not conceive. | Xs was accepted after 2 days by Tt and Qq. Xx sexually solicited Yy on the 4th day when Xs was re-introduced after 97-2# was removed temporally from Xx for a medical check*. The male was removed in late November, 1997. |
| 1998.7.10 | Xs | Tt birthed a female 98-4# on March 19 and Qq aborted* on February 18. Xx weaned 97-2# in September. | Qq sexually accepted Xs on the first day of introduction. Tt and Xx accepted him 3days later. Xs was removed on November 17, 1998. |
| 1999.7.13 | Xs | Xx aborted on February 21; Qq* aborted on March 28. Tt was nursing 98-4#. | Tt accepted Xs 2 days after his introduction. Xx and Qq sexually solicited Xs on the first day when he was introduced. Xs was removed from the cage on November 23, 1999. |
| 2000.7.9 | Yy | Qq* aborted on March 13; Tt aborted on March 20 and December 3. Xx aborted on April 22. | Yy was accepted after 7 days by the females. Yy became the full-time resident male. |
| 2001 | Yy | Qq produced a female 01-6# on April 2; Xx produced a male 01-3# on May 31; and Tt* aborted on December 16. |  |

* all the dead embryos were collected and none of the aborted females had a sick record at that time.
