# Supplementary material for: Female Resistance to Invading Males Increases Infanticide in Langurs
Source: PLoS One. 2011 Apr 22;6(4):e18971. doi: 10.1371/journal.pone.0018971 (PMC3081324; doi:10.1371/journal.pone.0018971)
Supplement: Table S2 — (DOC) [file pone.0018971.s002.doc]

**Table 2. Organizations of all OMUs and the changes of the Xiangguqing exhibition group of *R. bieti* after January 18, 2010**.

| OMU | OMU size | members | Notes |
| --- | --- | --- | --- |
| ZZ | 11 | 5Afs, 4SFs, 1I |  |
| SY | 11 | 5Afs, 5SFs |  |
| SZ | 8 | 3AFs, 1J, 3Is |  |
| HB | 6 | 3Afs, 3SFs, 1J |  |
| XM | 5 | 1Afs, 2SFs,1I | An SF joined from XSH on December 4, 2009. |
| XSH | 6 | 3Afs, 1SFs, 1J | Takeover occurred on December 4, 2009, and an SF left. |
| SanM | 8 | 3Afs, 4SFs |  |
| SiM | 8 | 4Afs, 2SFs,1J |  |
| JJ | 4 | 1Afs, 1SFs, 1J | The male abducted an AF from XM on January 18, 2010 to form this OMU. The same day an SF and a J joined in from XM. |

**Notes**: AF=adult female, SF=subadult female, J=juvenile, I=infant.
